# Community curation of bioinformatics software and data resources

**DOI:** 10.1093/bib/bbz075

**Published:** 2019-10-16

**Authors:** Jon Ison, Hervé Ménager, Bryan Brancotte, Erik Jaaniso, Ahto Salumets, Tomáš Raček, Anna-Lena Lamprecht, Magnus Palmblad, Matúš Kalaš, Piotr Chmura, John M Hancock, Veit Schwämmle, Hans-Ioan Ienasescu

**Affiliations:** 1 National Life Science Supercomputing Center, Technical University of Denmark, Building 208, DK-2800 Kongens Lyngby, Denmark; 2 Hub de Bioinformatique et Biostatistique – Département Biologie Computationnelle, Institut Pasteur, USR 3756 CNRS, Paris, France; 3 ELIXIR-EE, Institute of Computer Science, University of Tartu. J Liivi 2, Tartu, Estonia; 4 CEITEC - Central European Institute of Technology, Masaryk University Brno, Kamenice 5, 625 00 Brno-Bohunice, Czech Republic; 5 Faculty of Informatics, Masaryk University, Botanická 68a, 602 00 Brno, Czech Republic; 6 Department of Information and Computing Sciences, Utrecht University, Utrecht, Netherlands; 7 Center for Proteomics and Metabolomics, Leiden University Medical Center, Leiden, Netherlands; 8 Computational Biology Unit, Department of Informatics, University of Bergen, N-5020 Bergen, Norway; 9 Novo Nordisk Foundation Center for Protein Research, Faculty of Health and Medical Sciences, University of Copenhagen; 10 ELIXIR-Hub, Wellcome Trust Genome Campus, Hinxton, Cambridge, CB10 1SD, United Kingdom; 11 Department of Biochemistry and Molecular Biology and VILLUM Center for Bioanalytical Sciences, University of Southern Denmark, Campusvej 55, 5230 Odense, Denmark

**Keywords:** bioinformatics, software, database, registry, curation, community driven

## Abstract

The corpus of bioinformatics resources is huge and expanding rapidly, presenting life scientists with a growing challenge in selecting tools that fit the desired purpose. To address this, the European Infrastructure for Biological Information is supporting a systematic approach towards a comprehensive registry of tools and databases for all domains of bioinformatics, provided under a single portal (https://bio.tools). We describe here the practical means by which scientific communities, including individual developers and projects, through major service providers and research infrastructures, can describe their own bioinformatics resources and share these via bio.tools.

## Introduction

The corpus of bioinformatics resources is huge and expanding rapidly. Life scientists face a growing challenge in selecting tools that fit the desired purpose, especially cross-domain researchers who may be unfamiliar with expert terminology. To address this, the European Infrastructure for Biological Information (ELIXIR) [1] is supporting a systematic approach [2] towards a comprehensive registry of tools and databases for all domains of bioinformatics, provided under a single portal (https://bio.tools). bio.tools aims to provide, by a community-driven curation effort, concise and consistent metadata that are sufficient to inform end users about the main tool functionalities, to find and compare relevant software and to follow links where resources may be downloaded or used.

**Figure 1:**
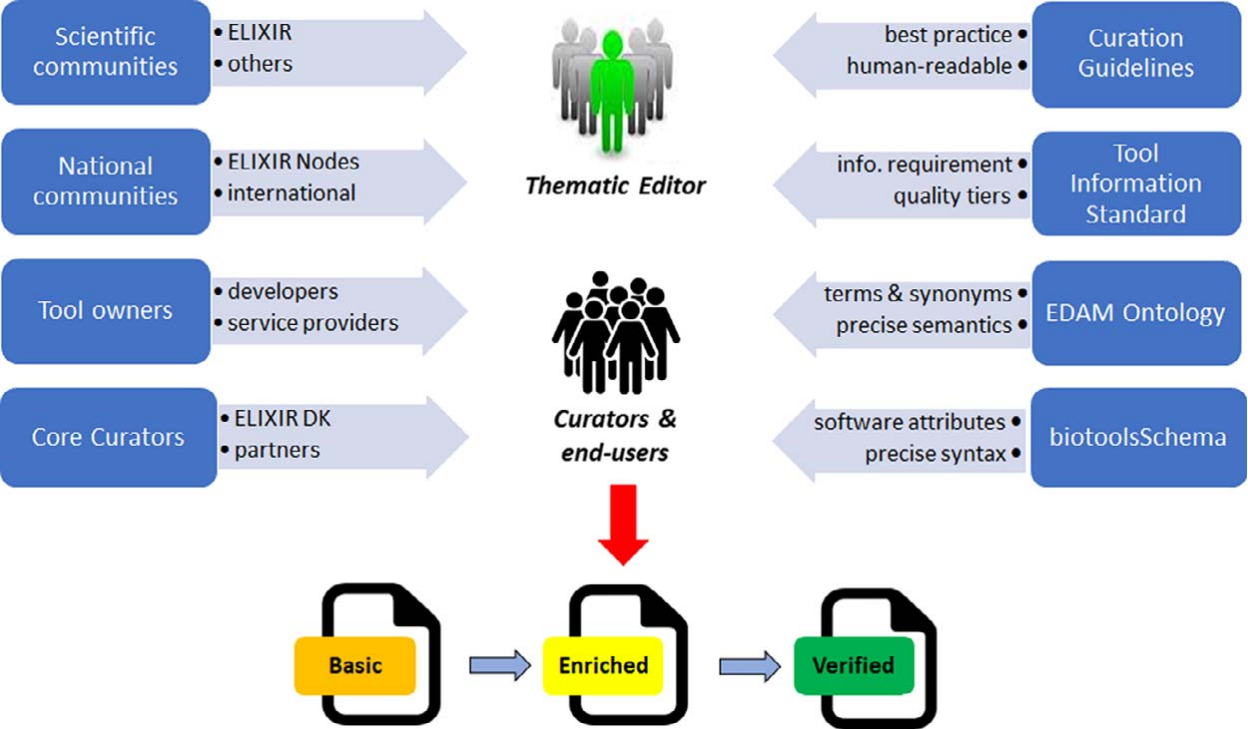
Foundation for resource curation. bio.tools depends upon individuals and communities (left of figure) and various technical components (right) to enrich and verify resource information. The effort is led by ELIXIR Denmark with oversight of Thematic Editors representing scientific and national communities.

**Table 1:** Resources for curation of software and database information

Resource	Description
bio.tools	Registry of life science software and databases bio.tools github.com/bio-tools/biotoolsRegistry/
biotoolsSchema	Formalized XML schema (XSD) for bioinformatics resource information github.com/bio-tools/biotoolsschema
EDAM ontology	Ontology of bioinformatics topics, operations, types of data, data identifiers and data formats github.com/edamontology/edamontology
Tool Information Standard	Standard for bioinformatics resource information requirement at various tiers of description richness bio-tools.github.io/Tool-Information-Standard
Ontology Lookup Service (OLS)	Ontology browser from EMBL-EBI www.ebi.ac.uk/ols/ontologies/edam
BioPortal	Ontology browser from NCBO bioportal.bioontology.org/ontologies/EDAM
EDAM Browser	EDAM browsing and development tool from IFB ifb-elixirfr.github.io/edam-browser github.com/IFB-ElixirFr/edam-browser
EDAMmap	Utility for text mining and mapping to EDAM ontology biit.cs.ut.ee/edammap/github.com/edamontology/edammap

**Table 2:** Links to documentation concerning bio.tools

Documentation	Description
bio.tools docs	Documentation for the bio.tools registry biotools.readthedocs.io/
Curators Guide	Human-friendly guidelines for writing bioinformatics resource descriptions biotools.readthedocs.io/en/latest/curators_guide.html
Thematic Editors Guide	Emerging guidelines for bio.tools Thematic Editors (see Community support and engagement) biotools.readthedocs.io/en/latest/editors_guide.html
API Usage Guide	Usage guidelines with examples for the bio.tools API biotools.readthedocs.io/en/latest/user_guide.html
API reference	Comprehensive reference information for the bio.tools API biotools.readthedocs.io/en/latest/api_reference.html
bio.tools—getting involved	Overview of ways to get involved with bio.tools biotools.readthedocs.io/en/latest/contributors_guide.html
biotoolsSchema docs	Documentation for the biotoolsSchema resource description model biotoolsschema.readthedocs.io
EDAM docs	Documentation for the EDAM ontology edamontologydocs.readthedocs.io
EDAM—getting involved	How to get involved with EDAM, including guidelines on how to request additions and changes edamontologydocs.readthedocs.io/en/latest/contributors_guide.html#requests
EDAM requests	Request additions and other changes to EDAM via GitHub (using documented issue templates or free-form requests) github.com/edamontology/edamontology/issues

A variety of platforms, often with overlapping scope but serving different audiences, aggregate or maintain tool and database information. The coordination and technical integration of bio.tools with these resources are at various levels of maturity. For several major institutional collections such as the ExPASy portal to bioinformatics tools and databases [3], tools developed by the IFB platform (https://www.france-bioinformatique.fr/en/services/tools) and EBI services [4], there is a common curation effort coordinated by ELIXIR. Collaborations around the sharing of data and expertise include also community projects such as the ms-utils.org Wiki (https://msutils.org) of software for analysis of mass spectrometry data, DebianMed [5] packages for medicine, pre-clinical research and life sciences and the Bioimage Informatics Search Engine (http://biii.eu/) for image analysis software. In the case of database metadata, there is scope for bio.tools and portals such as Identifiers.org [6] (an identifier resolution service for data collections) and FAIRSharing [7] (a portal for databases in context of standards and policies) to integrate their curation efforts, for example by co-maintaining a common set of descriptors in a public repository such as GitHub. In contrast, there are commercial portals (e.g. [8]) that aim to provide tailored solutions to paying customers.

The bio.tools initiative aims to foster individual tool developers, online service providers and scientific communities to share and curate their software productions to a common standard. To these ends, a rigorous foundation for software cataloguing (Figure 1, Tables 1 and 2) is being laid: biotoolsSchema—a general purpose description model for bioinformatics resources—defines a comprehensive list of common software attributes. It provides a rigorous structure and syntax allowing for software metadata validation. biotoolsSchema uses the EDAM ontology [9], which contains terms and synonyms for prevalent bioinformatics concepts, including types of data and data identifiers, data formats, operations and topics. It provides rigorous semantics for description of scientific aspects. An emerging Tool Information Standard, based on biotoolsSchema and EDAM, describes what attributes should be provided at various tiers of detail and quality. The standard refers to bio.tools Curation Guidelines, which specify how tool information should be specified: stylistic or other aspects that cannot conveniently be expressed in a formal schema or ontology.

While a technical foundation is doubtlessly required, the problem of curation remains fundamentally social; a diligent manual effort is required to ensure the corpus of resource descriptions is reasonably comprehensive and is kept up to date with new tools or changes to existing ones. The most challenging part, especially given the complexity of modern software and scientific disciplines that constantly evolve, is providing the practical annotation of tool scientific function. Expert understanding is needed to get this right: for ontology development, individual tool annotation and catalogue-wide consistency of curation. In the absence of large funding of such activities, efforts such as that on bio.tools must leverage the goodwill and expertise of the community in order to succeed.

We describe here the practical means by which scientific communities, including individual developers and projects, through major service providers and research infrastructures, can describe their own software and database productions and share these via bio.tools. The paper is structured as follows. We begin with some general considerations and a summary of sources of resource information. We then summarize biotoolsSchema and EDAM in the context of the Tool Information Standard and key considerations such as the annotation of tool function. Various methods and utilities for resource annotation are presented, as works in progress, with a note on how these can be applied to the description of one or a few tools or to larger collections. We then outline the steps involved in the curation of a corpus of resources and how they can be tailored to suit a specific community, before sketching future challenges and possible directions. The work is led by ELIXIR Denmark in context of a broader ELIXIR initiative (https://www.elixir-europe.org/communities) that aims to foster scientific communities and support them to integrate their activities within the ELIXIR infrastructure.

### General considerations

Each producer of bioinformatics resources, whether a nation, institute, lab or scientific community, has distinctive requirements and technical and scientific expertise that can brought to bear most efficiently during *en masse* curation of resource in their specialized area. The curation of a corpus of tools into bio.tools involves a multi-step process and must be carefully planned. Enumeration of tools known to be important is followed by mining of the web and scientific literature for relevant information. Annotation of scientific features (using EDAM) and more general attributes (defined in biotoolsSchema) should proceed systematically and follow patterns from the Information Standard and Curation Guidelines. Most importantly, the priorities should reflect those resources and specific types of information that are important to a community. Where ontology development is required, this too has to proceed in a coordinated way. Due care is needed, to understand the model of tool function used by bio.tools and to ensure an appropriate level of detail is specified and that details are relevant to the latest available tool version.

### Sources of information

Various sources of information can be used when collating and annotating a corpus of tools:

(i) lists of tools and types of tool information that are key to a community and can be ascertained, for example, by user survey
(ii) collections of tools already registered in bio.tools, annotated with one or more EDAM topics and possibly tagged as belonging to a named collection [searches are provided by the bio.tools user interface (UI)]
(iii) the primary publication that describes the tool
(iv) the official website of the tool and other textual materials such as user manual, supplementary files, tutorials, etc*.*
(v) source code and technical documentation available from an online repository (in the case of open source tools)
(vi) informal information, such as tips and hints from colleagues, especially concerning tool alternatives, newer versions, usage trends and so on

Particular attention should be given to those tools and types of information that are of relevance to tool end users, which vary from community to community. Texts highly descriptive of a tool’s operations, inputs and outputs should be recorded, as these are helpful, later, when annotating the tool’s functionality using EDAM.

### Resource descriptors

biotoolsSchema defines the data model used to describe the resources included in bio.tools. It defines general, scientific and technical attributes for resource description and includes a simplified model of tool function that makes use of the EDAM ontology for the scientific description of tool inputs, output and operations. These aspects are summarized below.

#### biotoolsSchema

biotoolsSchema defines some 50 general software attributes such as name, description and homepage URL. It provides, through regular expression patterns and 16 controlled vocabularies, a precise syntax and nomenclature for the general characterization of tools, including:

(i) Tool type*—*the type of application software, e.g. ‘Command-line tool’
(ii) Language—name of programming language the software source code was written in, e.g. ‘C’
(iii) Operating system*—*the operating system supported by a downloadable software package, e.g. ‘Linux’
(iv) Accessibility—whether the software is freely available for use, e.g. ‘Open access’
(v) License*—*software or data usage license, e.g. ‘GPL-3.0’
(vi) Download type—type of download that is linked to, e.g. ‘Source code’
(vii) Link type—the type of data, information or system that is obtained when the link is resolved, e.g. ‘Helpdesk’
(viii) Documentation type*—*type of documentation that is linked to, e.g. ‘API documentation’
(ix) Version—Version information (typically a version number) of the software applicable to the *bio.tools* entry, e.g. ‘1.0’

biotoolsSchema is supported by the bio.tools API and UI for registration purposes (see Curation tooling below). It supports metadata specification in a choice of serialization formats (XML and JSON currently) and can (through widely and freely available tools) be used to validate tool descriptions prior to registration in bio.tools. This is particularly useful where a large number of tools are being collated and described, before *en masse* registration*.* biotoolsSchema is made freely available and is comprehensively documented. In case biotoolsSchema does not model the information required by a particular community or use-case, extensions to the model can be requested via GitHub (Table 1).

#### EDAM

The EDAM ontology contains terms and synonyms for prevalent bioinformatics concepts, including research area and task-specific attributes, which provides a precise nomenclature for functional characterization of tools:

(i) topic*—*a broad category within the life sciences to which the tool is applicable, e.g. ‘Proteomics’
(ii) operation—specifically what a tool does, e.g. ‘Peptide identification’
(iii) data*—*types of input and output data, e.g. ‘Mass spectrometry spectra’.
(iv) identifier (included under data)—a specific type of identifier of some data record, e.g. ‘Uniprot accession’
(v) format*—*specific data formats supported by the tool, e.g. ‘Thermo RAW’

EDAM is freely available and comprehensively documented (Table 2). EDAM can be searched and browsed at various online browsers (Table 1), each providing complementary functions. In case new concepts, synonyms of existing terms or other changes are needed, these may be proposed via GitHub after first reading the guidelines for making requests (Table 2). Requests can also be made using the EDAM Browser tool (see Curation tooling). In case many additions or changes are required, there may well be a more efficient way to proceed, and it is advisable to discuss the way to proceed first with the EDAM developers. EDAM is an open, community-driven project, and new contributors are most welcome to join the project.

#### Model of tool function

**Figure 2:**
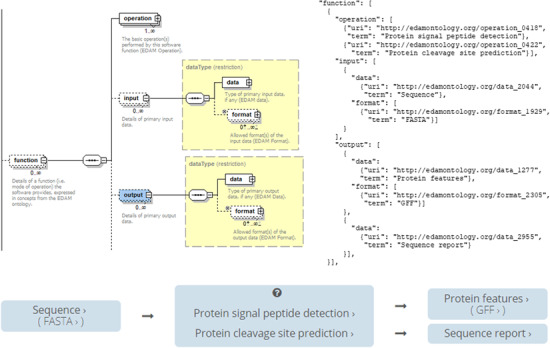
Model of the tool function in bio.tools as illustrated for signalp (biotools:signalp) [10]. The basic model is illustrated alongside the JSON representation and how the tool function appears in the bio.tools Tool Card.

bio.tools uses a model of tool function (Figure 2) defined within biotoolsSchema. A tool can have one or more basic functions (modes of operation), each function performing one or more specific operations. In turn, an operation may have one or more primary inputs and outputs, each of a defined type of data and listing supported format(s). bio.tools does not mandate that all of this information is specified; however, careful consideration of the level of required detail is recommended before annotation commences. In the simple use case of describing tools to make them more discoverable, it may suffice to simply annotate the major operations performed by the tool. In the more challenging case of tool interoperability, explicit annotation of data types and formats may be required. In any case, the suggested annotation processes are:

(i) Identify the distinct functions (modes of operation) and the individual operations associated with each one. Typically, different functions (modes) perform different operations, and for well-documented tools this is usually obvious.
(ii) As a rule, if the tool allows an option between doing one thing or another, then the operations should be assigned to distinct functions. If in contrast a tool always does one or more things, annotate these as distinct operations within a single function.
(iii) bio.tools aims for fairly coarse-grained descriptions of functionality, and it is recommended (depending on the use case) to only specify the primary functions and operations, from a typical end-user perspective. If a tool happens to perform some operation internally, but this is secondary to its advertised purpose, then it should not be annotated.
(iv) The above point holds for input and output too, e.g. a sequence alignment tool would be annotated as reading sequences (input) and writing a sequence alignment (output), but not (typically) with gap insertion and extension penalties, or other parameters.

Further advice is available in the Curators Guide (see Tool information standards below) and via the bio.tools helpdesk (see Getting involved and support below).

#### Tool Information Standards

The Tool Information Standard used by bio.tools defines five tiers (from ‘Sparse’ to ‘Comprehensive’) of progressively richer annotation that may be provided for a resource. For example, the ‘Basic details’ tier mandates annotations for resource name, description, homepage, unique ID, tool type, scientific topic, publication and support. The standard is based on biotoolsSchema and EDAM. For the ‘Basic details’ example, ‘scientific topic’ is satisfied by an EDAM Topic annotation, and ‘support’ may be satisfied by any one of a link to a helpdesk, issue tracker or mailing list or by contact details (URL or email) for a person. The standard proposes a general purpose information requirement and provides a framework to guide the curation tasks and priorities. In practice, one is not bound by it, but should select and prioritize those aspects that are most relevant to the community in question.

**Figure 3:**
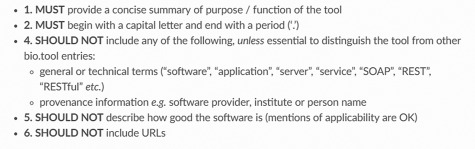
An example guideline (for tool ‘description’—textual description of the software) from the bio.tools Curators Guide.

Accompanying the standard, there are bio.tools Curation Guidelines that describe how to create a high-quality tool description, above and beyond the syntactic and semantic constraints that are defined in biotoolsSchema and EDAM. The Curators Guide includes some general guidelines for example on the use of EDAM, as well as guidelines for specific attributes (Figure 3) and types of tool defined in biotoolsSchema. Again, bio.tools does not enforce the guidelines, but it is recommended to adapt and use them for the purpose at hand.

### Curation tooling

Several interfaces within bio.tools and standalone utilities (described below) are available to assist the curator, and these can be used before and during the registration of tools in bio.tools.

#### bio.tools

Anyone with a bio.tools account can create new entries, edit existing entries and share edit rights with or transfer entry ownership to other users. There are two tool registration options:

(i) manual registration via the bio.tools registration UI
(ii) programmatic registration via API by submission of a file in JSON or XML format.

In either case, to be saved successfully, tool details provided must conform to the software description model as defined by biotoolsSchema and encapsulated by bio.tools. The validation goes beyond what is encoded within biotoolsSchema, ensuring for example that the supplied tool name is unique for purposes of producing a valid tool identifier and that EDAM concept URIs are not deprecated (i.e. marked in EDAM as obsolete, which happens occasionally during ontology revisions).

**Figure 4:**
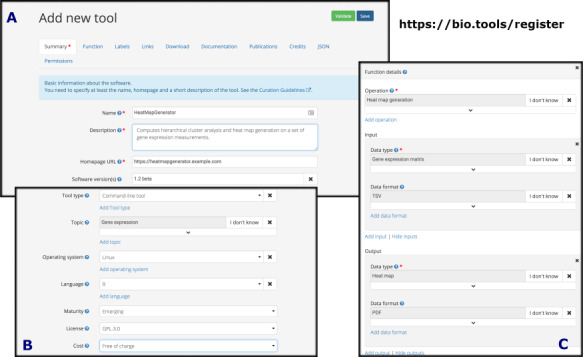
The bio.tools registration interface provides convenient widgets for creating and editing tool descriptions for registration in bio.tools. The entry page (A) and ‘Labels’ (B) and ‘Function’ (C) tabs are shown.

The bio.tools registration interface (Figure 4) simplifies the creation and editing of valid registry entries and is available to logged-in users. A simple widget assists with EDAM annotations; it displays the EDAM ontology as a tree and allows a user to browse and search for relevant terms. The functionality is currently rather limited compared to the more fully fledged EDAM browsers.

**Figure 5:**
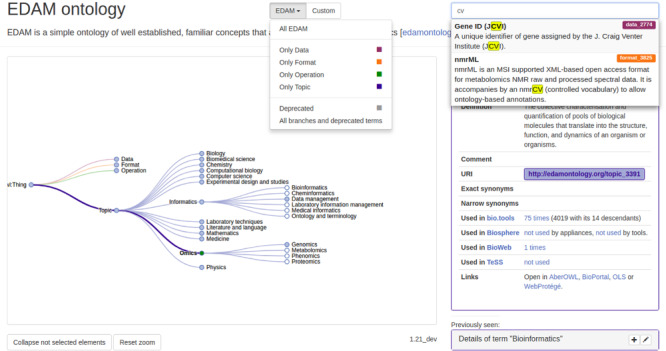
EDAM Browser allows for browsing and contribution to EDAM through a convenient tree view and an EDAM request templating function. An information panel shows information about EDAM concepts and their usage in various contexts.

The choice of tool registration via UI or API depends upon the curation method. The UI is most suitable for the registration of one or a few tools, by non-technical end users. In case information on many tools has been collated collaboratively, for example using a Google spreadsheet, transformation to biotoolsSchema-compatible XML, followed by validation and use of the API, may be more convenient. Comprehensive documentation including an API reference and usage guide are available (Table 2).

#### EDAM Browser

EDAM Browser [11] (Figure 5) is a standalone web application tailored specifically to the structure and properties of EDAM, which assists EDAM browsing, term selection and understanding of term usage in various contexts. It targets both contributors and users of bio.tools and EDAM who might not be ontology experts. EDAM can be explored by browsing the interactive tree representation of the ontology or by using the autocomplete bar that searches over all EDAM concept properties. EDAM Browser displays in a side panel, key information for the selected concept, including its properties, ancestors and non-hierarchical relations to other concepts (e.g. the expected inputs and outputs of an operation as stated in EDAM). Importantly, the browser provides a live display of the number of annotated resources and links to various databases that use this term, including bio.tools, IFB’s BioSphere cloud (https://biosphere.france-bioinformatique.fr/) and TeSS [12]. Furthermore, any user may directly request a modification on an existing concept or the addition of a new concept, via a form that gathers all necessary information and directly formats the suggestion as an issue ready to be submitted by the user to EDAM GitHub issue tracker. EDAM Browser has a plug-in framework, allowing links to new databases with usage counts to be easily set up. The whole system and its components, such as autocomplete search bar and the tree visualization, are reusable and could easily be integrated into external websites and applications.

#### EDAMmap

**Figure 6:**
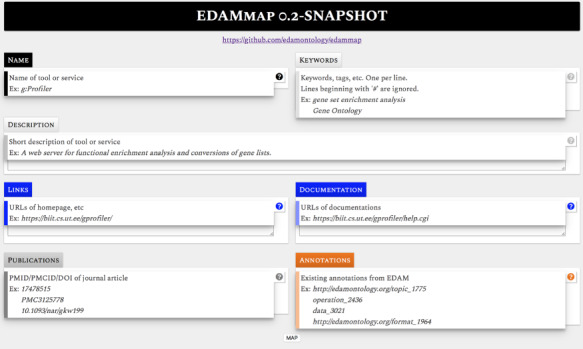
EDAMmap helps a curator by providing candidate EDAM annotations for tools and databases, generated automatically from text mining a variety of sources of resource information.

EDAMmap is a standalone utility designed to help curators, by suggesting appropriate EDAM annotations for a resource that is to be added to bio.tools. The suggestions are based on mining a variety of textual sources such as web pages, publications and free text of any length. It is available as a web application (Figure 6) and a command-line tool. Free text from the specified sources is tokenized and mapped to the EDAM ontology terms, and the most likely appropriate EDAM terms are outputted. The web application includes input fields for keywords, description, links (e.g. homepage), documentation, publications and already existing EDAM annotations (which help in case a user already knows some relevant annotations). Documents are specified by URL and publications by PMID, PMCID or DOI identifiers. The resource name and at least one source of information are mandatory. Several parameters can be adjusted, enabling users to find settings that are most appropriate for the information they have in hand. By default, EDAMmap outputs three suggestions for each of the EDAM Topic and Operation branches, but this can be adjusted, along with the parameters of the mapping algorithm and weights of different information sources. The command line version requires information sources to be specified in a CSV file and can output suggestions for many resources at a time. Accurate annotation, of course, relies heavily on expert domain knowledge, and EDAMmap is intended to support but cannot replace the curator.

Several other utilities are under active development (see http://github.com/bio-tools/) and promise to improve the curation process and reuse of bio.tools data. We highlight a few here to encourage participation in these open projects:

(i) edamToolAnnotator (https://github.com/bio-tools/edamToolAnnotator) is a utility for annotating a tool using EDAM terms. It will eventually replace the corresponding functionality in the bio.tools registration interface.
(ii) bio.tools Sum (https://github.com/bio-tools/biotoolssum) is a client-side web application for rendering reports on local websites of tool collections from bio.tools. A working example customized for tools and services from the ELIXIR Czech node is available. The utility can be customized for use by other sites and communities.
(iii) ReGaTE [13] (https://github.com/C3BI-pasteur-fr/ReGaTE) is a command line utility that facilitates the registration of the tools installed on any given Galaxy portal in bio.tools. As a future work we will improve the method, to enable the linking of multiple servers providing a given tool as Galaxy-based services, to a single bio.tools entry.
(iv) ToolDog [14] (https://github.com/bio-tools/ToolDog) is a utility that uses bio.tools to generate tool descriptions for use in workbench environments including Galaxy [15] and Common Workflow Language (https://www.commonwl.org/).

### Curation process

The curation process should be tailored to the specific requirements, expertise and capacity of the community in question, so it is impossible to prescribe a process that works for everyone. Curation can involve editing existing bio.tools entries as well as creating new ones. A few broad pointers follow.

(i) Enumerate commonly used software and databases*.* A comprehensive list of key resources should be compiled including tools already in bio.tools, specialized registries and Wikis or lists of tools from the Web. This can be augmented by searches of the Web and the scientific literature. The minimum information requirement (tool name, homepage URL and short description) of bio.tools barely presents a barrier to new registrations, and it is recommended to use bio.tools from the outset, when compiling the list. An alternative is co-editing a shared spreadsheet. In any case, the DOI of the tool’s primary publication (where available) should be recorded, as this is very useful for later annotations.
(ii) Decide on information requirement and curation priorities*.* The information requirement depends upon the exact use case. It is recommended to enumerate what software attributes are important and prioritize these, for example, by triaging the list of 50 attributes defined by biotoolsSchema into ‘essential’, ‘important’ and ‘nice to have’; these priorities can be set once the needs of individual users or a community are understood, for example from a user survey. For general purposes, one of the tiers in the standard (see Tool information standards) may be applicable, and in which case curation can proceed in iterations delivering progressively more details as per the tiers.
(iii) First curation pass. From experience of constructing bio.tools, many software attributes are obvious from a quick (10 min, say) inspection of the tool publication and homepage. This includes, for example, contact details, repository URL, license, relevant topics and operations performed by the tool. An intensive ‘curatathon’ to quickly harvest these low-hanging fruit is recommended. Where EDAM annotations cannot immediately be assigned, passages or sentences describing the tool functionality should be recorded for the annotation of tool function (see Annotation of tool function below).
(iv) Further curation passes. Tool metadata can be progressively enriched, following tiers in the Tool Information Standard or the priorities established earlier, by a deeper inspection of the available sources of information. Various utilities (see Curation tooling) can assist with this. It is strongly recommended, by this stage, to edit bio.tools entries, allowing end users of the data to easily validate and verify the curation process.
(v) Annotation of tool function. In case detailed annotation of tool function is required, various sources may be used for deciding on the assignment or creation of new EDAM operation, data or format terms. The EDAMmap utility can help identify relevant terms in the sources. Ideally, tool annotations should be assigned independently and cross-validated, to iron out any disagreements and arrive at a reliable, consensus description of a tool. Once the corpus of tools is complete, inspection of EDAM annotations across the entire tool collection will allow for final adjustments, ensuring consistency and a definitive controlled vocabulary.
(vi) EDAM developments*.* It is recommended to work with the EDAM developers to ensure any required new terms are added in a timely manner. At the same time, EDAM can be extended, not only with the required new concepts (terms) but also with synonyms of existing concepts (to improve tool findability in various contexts) and various other changes to improve EDAM conceptual hierarchy and usability.
(vii) Wrapping up*.* Tool descriptions can be polished and updated post-registration using the bio.tools editing interface. For this purpose, editing rights on the entries can be shared with anyone, or credentials for a single bio.tools account can be shared among a trusted group of users.

The above process should work in general but should be optimized depending upon specific circumstances around the community and existing coverage in EDAM and bio.tools. For example, if very few relevant tools are registered in bio.tools and semantic coverage in EDAM is very patchy, or the converse, a different approach may well be merited, and it is recommended to discuss this first (mail registry-support@elixir-dk.org in the first instance).

### Community support and engagement

Maintaining a corpus of tool descriptions in the long term depends upon effective community engagement. bio.tools offers direct assistance to individual developers of tools or providers of online services, as well as to organizations that foster a community, for example by participating in community-led curation events. ELIXIR is establishing a network of Thematic Editors, experts within fields of the life sciences who are motivated to liaise with their respective communities (national or scientific) and provide a bridge to bio.tools, supporting developments tailored to that community. In this context, the Danish ELIXIR node ran a studentship scheme, to support early career stage scholars, working under the aegis of a Thematic Editor, to contribute to bio.tools and gain experience with the ELIXIR infrastructure. This mechanism has proved to be an efficient method for bulk curation work. These initiatives are at an early stage, and your involvement is most welcome; more information is available online (Table 2).

The work above is in context of a broader ELIXIR initiative (https://www.elixir-europe.org/communities) to foster communities and bring together experts to develop standards, services and training within specific life science domains. ELIXIR Communities are international groupings of experts in a particular technical or scientific area, intended to drive the technical evolution of ELIXIR. Communities hold a special place in ELIXIR because they can receive funding from ELIXIR: for activities such as annual workshops and staff exchange and through ELIXIR Implementation Studies (relatively small projects, funded over 2 years, that drive the development of the ELIXIR infrastructure). ELIXIR recently announced four new community-led Implementation Studies that bring together Communities with the ELIXIR Platforms https://elixir-europe.org/news/new-portfolio-community-led-implementation-studies-selected). ELIXIR currently recognizes eight Communities: Human Genomics Translational Data, Rare Diseases, Human Copy Number Variation, Crops and Forest Plants, Marine Metagenomics, Proteomics, Metabolomics and Galaxy. A number of other communities have indicated an interest in becoming part of ELIXIR and are in the process of being considered for approval. ELIXIR Communities are ideal resources to draw upon for subject-specific annotation and have been, and will continue to be, drawn upon for this purpose.

bio.tools and the other open projects and initiatives described here welcome your involvement. Information and instructions for new contributors are available online (Table 2) for bio.tools and EDAM and include details of mailing lists, how to make suggestions and requests, tasks and feature management, forthcoming meetings and events and so on. Direct assistance with bio.tools is available by emailing registry-support@elixir-dk.org. The preferred option for communication, and especially for bug reports and suggestions, is GitHub (http://github.com/bio-tools/biotoolsregistry/).

## Discussion

We have summarized, as a work in progress, the means by which software and databases can be described and shared via bio.tools, putting this in context of the various open projects and community-driven initiatives that are being fostered by the ELIXIR infrastructure. Such efforts provide the best hope for the sustainable provision and maintenance of high-quality software information in the long term, required for various contexts and use cases. Beyond merely improving the findability of tools and the dissemination of basic information, the data have exciting applications, for example, in the automated construction and evaluation of alternative bioinformatics pipelines [16, 17]. Such applications are only possible if carefully assigned functional annotation is available. To both ends, much work remains to be done and will include production of ‘gold standard’ tool descriptions for specific communities, provision of the bio.tools data in linked open data formats and integration of bio.tools with other products such as Biocontainers.pro [18], Galaxy and EuropePMC [19], to combine the bio.tools data with information about where tools can be used or downloaded in an executable form and put in deeper context of their scientific application. There is also a need to promote better information standards for life science software more generally, such as we have described for EDAM, biotoolsSchema and the Tool Information Standard. All the software described here and the bio.tools data itself are made available under open license. We welcome contributions and collaborations in all areas to improve the corpus of bioinformatics tool descriptions for the benefit of Life Scientists everywhere.

## Notes

Key Points

- The bio.tools registry enables software developers and service providers to create and share descriptions of their resources.
- Resource descriptions adhere to a consistent and rigorous syntax and semantics, including controlled vocabulary terms describing tool operations, inputs and outputs.
- Support is provided to help individuals and research communities contribute to bio.tools.

## Notes

**Jon Ison** is a Senior Scientist working at the Technical University of Denmark, one of the leading research and education institutions in Europe for the technical and natural sciences.

## Notes

**Hervé Ménager** is a research engineer at Institut Pasteur where he leads a software development team focusing on Web technologies for publishing and sharing scientific tools, analysis, data and workflows.

## Notes

**Bryan Brancotte, Erik Jaaniso, Ahto Salumets and Tomáš Raček** are software developers and long-standing contributors to bio.tools.

## Notes

**Anna-Lena Lamprecht** is an Assistant Professor in the Software Technology group at the Department of Information and Computing Sciences of Utrecht University, and works in the areas of scientific workflows and applied computational science.

## Notes

**Magnus Palmblad** heads the Bioinformatics Research Group at the LUMC, working in the area of proteomics bioinformatics and the annotation and dissemination of open source software for mass spectrometry data analysis.

## Notes

**Matúš Kalaš** is a postdoctoral fellow at the Department of Informatics of Bergen University and is a long-standing contributor to bio.tools and the EDAM ontology.

## Notes

**Piotr Chmura** is a research programmer and long-standing contributor to bio.tools, working in the Brunak Group whose focus is translational disease systems biology.

## Notes

**John Hancock** is the Communities and Services Coordinator for the ELIXIR research infrastructure and has a long history in research and management positions in computational biology in the UK.

## Notes

**Veit Schwämmle** is an Associate Professor at SDU where he leads the Computational Proteomics Group, which develops and applies computational solutions for improved data analysis in large-scale omics experiments.

## Notes

**Hans-Ioan Ienasescu** works at the Technical University of Denmark and is the lead software developer of bio.tools.

## References

[1] Crosswell LC and Thornton JM. ELIXIR: a distributed infrastructure for European biological data. Trends Biotechnol 2012;30(5): 241–2, doi: 10.1016/j.tibtech.2012.02.002. 22417641

[2] Ison J, Rapacki K, Ménager H, et al. Tools and data services registry: a community effort to document bioinformatics resources. Nucleic Acids Res 2015;44(D1):D38–47, doi: 10.1093/nar/gkv1116. 26538599 PMC4702812

[3] Artimo P, Jonnalagedda M, Arnold K, et al. ExPASy: SIB bioinformatics resource portal. Nucleic Acids Res 2012;40(W1):W597–603, doi: 10.1093/nar/gks400. 22661580 PMC3394269

[4] Park YM, Squizzato S, Buso N, et al. The EBI search engine: EBI search as a service—making biological data accessible for all. Nucleic Acids Res 2017;45(W1):W545–W549, doi: 10.1093/nar/gkx359. 28472374 PMC5570174

[5] Möller S, Krabbenhöft HN, Tille A, et al. Community-driven computational biology with Debian Linux. BMC Bioinformatics 2010;11(S12):S5, doi: 10.1186/1471-2105-11-s12-s5. PMC3040531 21210984

[6] Juty, N, Le Novère N, Hermjakob H, et al. Towards the collaborative curation of the registry underlying Identifiers.org. Database (Oxford) 2013;2013:bat017, doi: 10.1093/database/bat017. 23584831 PMC3625955

[7] Sansone S-A, McQuilton P, Rocca-Serra P., et al. FAIRsharing as a community approach to standards, repositories and policies. Nat Biotechnol 2019;37(4):358–367, doi: 10.1038/s41587-019-0080-8. 30940948 PMC6785156

[8] Henry VJ, Bandrowski AE, Pepin A-S, et al. OMICtools: an informative directory for multi-omic data analysis. Database (Oxford) 2014;2014:bau069, doi: 10.1093/database/bau069. 25024350 PMC4095679

[9] Ison J, Kalaš M, Jonassen I, et al. EDAM: an ontology of bioinformatics operations, types of data and identifiers, topics and formats. Bioinformatics 2013;29(10):1325–32, doi: 10.1093/bioinformatics/btt113. 23479348 PMC3654706

[10] Nielsen, Henrik. Predicting secretory proteins with SignalP. Methods Mol Biol 2017;1611:59–73, doi: 10.1007/978-1-4939-7015-5_6. 28451972

[11] Brancotte B, Blanchet C, Ménager H. A reusable tree-based web-visualization to browse EDAM ontology, and contribute to it. J Open Source Softw 2018;3(27):698, doi: 10.21105/joss.00698.

[12] Larcombe L, Hendricusdottir R, Attwood TK, et al. ELIXIR-UK role in bioinformatics training at the national level and across ELIXIR. F1000Res 2017;6:952, doi: 10.12688/f1000research.11837.1. PMC5521157 28781748

[13] Doppelt-Azeroual O, Mareuil F, Deveaud E, et al. ReGaTE: Registration of Galaxy Tools in Elixir. GigaScience 2017;6(6):1–4, doi: 10.1093/gigascience/gix022. PMC5530318 28402416

[14] Hillion KH, Kuzmin I, Khodak A, et al. Using bio.tools to generate and annotate workbench tool descriptions. F1000Res 2017;6:2074, doi: 10.12688/f1000research.12974.1. PMC5747335 29333231

[15] Afgan E, Baker D, Batut B, et al. The galaxy platform for accessible, reproducible and collaborative biomedical analyses: 2018 update. Nucleic Acids Res 2018;46(W1):W537–44, doi: 10.1093/nar/gky379. 29790989 PMC6030816

[16] Lamprecht AL, Naujokat S, Margaria T, et al. Semantics-based composition of EMBOSS services. J Biomed Semantics 2011;2(Suppl 1):S5, doi: 10.1186/2041-1480-2-s1-s5. PMC3105497 21388574

[17] Palmblad M, Lamprecht AL, Ison J, et al. Automated workflow composition in mass spectrometry-based proteomics. Bioinformatics 2018;35(4):656–64, doi: 10.1093/bioinformatics/bty646. PMC6378944 30060113

[18] da Veiga LF, Grüning BA, Alves Aflitos S, et al. BioContainers: an open-source and community-driven framework for software standardization. Bioinformatics 2017;33(16):2580–2, doi: 10.1093/bioinformatics/btx192. 28379341 PMC5870671

[19] The Europe PMC Consortium. Europe PMC: a full-text literature database for the life sciences and platform for innovation. Nucleic Acids Res 2014;43(D1):D1042–8, doi: 10.1093/nar/gku1061. 25378340 PMC4383902

